# “Who Will Marry a Diseased Girl?” Marriage, Gender, and Tuberculosis Stigma in Asia

**DOI:** 10.1177/1049732318812427

**Published:** 2018-11-30

**Authors:** Bethan Hatherall, James N. Newell, Nick Emmel, Sushil Chandra Baral, Muhammad Amir Khan

**Affiliations:** 1 University of East London, London, United Kingdom; 2 University of Leeds, Leeds, United Kingdom; 3 HERD International, Kathmandu, Nepal; 4 Association for Social Development, Islamabad, Pakistan

**Keywords:** tuberculosis, stigma, Bangladesh, Pakistan, Nepal, qualitative research, grounded theory

## Abstract

In a qualitative study on the stigma associated with tuberculosis (TB), involving 73 interviews and eight focus groups conducted in five sites across three countries (Bangladesh, Nepal, and Pakistan), participants spoke of TB’s negative impact on the marriage prospects of women in particular. Combining the approach to discovering grounded theory with a conceptualization of causality based on a realist ontology, we developed a theory to explain the relationships between TB, gender, and marriage. The mechanism at the heart of the theory is TB’s disruptiveness to the gendered roles of wife (or daughter-in-law) and mother. It is this disruptiveness that gives legitimacy to the rejection of marriage to a woman with TB. Whether or not this mechanism results in a negative impact of TB on marriage prospects depends on a range of contextual factors, providing opportunities for interventions and policies.

## Introduction

Tuberculosis (TB), an infectious disease caused by the bacillus *Mycobacterium tuberculosis*, causes ill-health for an estimated 10 million people worldwide each year, 45% of whom are in South and East Asia (World Health Organization [WHO], 2017). Without treatment, the mortality rate from TB is high, but with a timely diagnosis and correct treatment (typically a minimum 6-month drug regimen), most people who develop TB can be cured (WHO, 2017). However, the economic and social consequences of having TB disease and of seeking TB care can be substantial (Courtright & Turner, 2010; Tanimura, Jaramillo, Weil, Raviglione, & Lönnroth, 2014).

Across South Asia, TB has been associated with social stigma (Balasubramanian, Oommen, & Samuel, 2000; Baral, Karki, & Newell, 2007; Liefooghe, Michiels, Habib, Moran, & De Muynck, 1995; Nair, George, & Chacko, 1997; Somma et al., 2008; Weiss, Auer, Somma, & Abouihia, 2006). To effectively reduce stigma, Link and Phelan (2001) have argued that its fundamental causes need to be addressed. While explanations for TB stigma have focused on fear of infection, perceived associations of TB with other disvalued characteristics, and on the belief that those with TB are to blame for their infection (Courtright & Turner, 2010), knowing whether these constitute fundamental causes is complicated by the tendency in research on stigma to conflate causes, functions, and effects (Deacon et al., 2005). Furthermore, as a construct, stigma is increasingly being seen as multidimensional, requiring potentially different and distinct causal theories and intervention approaches for each of its dimensions (Clair, Daniel, & Lamont, 2016; Pescosolido, 2015). Macintyre et al. (2017) call for research to provide greater clarity on what causes a dimension of TB stigma to emerge and thrive in different contexts and populations.

The adverse impact of TB on marriage prospects has previously been identified as a dimension of TB stigma (Courtright & Turner, 2010). Indeed concerns over marriage prospects, particularly for women, following a diagnosis of TB have been reported in numerous studies conducted across South Asia (Atre, Kudale, Morankar, Gosoniu, & Weiss, 2011; Baral et al., 2007; Ganapathy et al., 2008; Liefooghe et al., 1995; Nair et al., 1997; Sagili, Satyanarayana, & Chadha, 2016; Somma et al., 2008). Policies or interventions to address the adverse impact of TB on marriage prospects, whether experienced or anticipated, need to be underpinned by a causal theory to explain the relationships between TB, marriage, and gender. As part of a wider multicountry qualitative study on TB stigma, we developed such a causal theory using a realist conceptualization of causality (Maxwell, 2012; Pawson & Tilley, 1997). From a realist perspective, causality in the social world involves a combination of an underlying mechanism and the context enabling this mechanism to generate a certain outcome, such as the negative impact of TB on marriage prospects. While identifying the underlying mechanism is a crucial step in understanding why TB impacts on marriage prospects, the contexts that influence whether or not the mechanism becomes generative are equally crucial to this understanding. In this article, we describe the development of our realist causal theory and ask how, for whom, and in which circumstances does TB affect marriage prospects.

## Method

The grounded theory method is a rigorous approach to developing theories through the iterative processes of qualitative data collection, coding, and analysis (Glaser & Strauss, 1967). We chose the approach as the overall aim of our research was to develop explanatory theories of dimensions of TB stigma, one of which we identified as being the negative impact of TB on marriage prospects, particularly for women. Furthermore, the philosophical orientation underpinning the grounded theory approach, namely, symbolic interactionism, aligns with our conceptualization of stigma as a social construct, produced, resisted, and negotiated through interactions between people.

Researchers at the Nuffield Center for International Health and Development at the University of Leeds led the study in collaboration with teams of researchers from the Health Research and Social Development Forum (HERD) in Nepal, the Association for Social Development (ASD) in Pakistan, and the National TB Control Program in Bangladesh. Bangladesh, Nepal, and Pakistan were chosen for this study of TB stigma in South Asia because the Nuffield Center had previous experience of conducting collaborative research on TB in these three Asian countries (Newell, Baral, Pande, Bam, & Malla, 2006; Walley, Khan, Newell, & Khan, 2001; Ullah et al., 2012). Ethical approval for the study was granted by the University of Leeds research ethics committee (February 8, 2007), by the National Bioethics Committee in Pakistan (Ref. F.4-87/NBC/ASD-Project/10/5404), by the Institute of Medicine at Tribhuvan University in Nepal (February 13, 2007), and by the National TB Control Program in Bangladesh (Memo No. 5-15/TB-2EP/TB Research/04-07/10253).

A 3-day workshop was held in Pakistan, at the start of the study, for all the researchers involved to meet each other and discuss the research topic, design, and process. This was followed by a period of preparation in which the research teams (comprising 2–3 people per team) in each of the three countries were provided with bespoke contextually sensitive support and training in qualitative research methods and ethical research conduct in their respective countries. The training was provided by this article’s first author, Bethan Hatherall, who has a background in social and public health research.

### Site Selection

Heterogeneity of sites (e.g., urban and rural) and populations within sites (e.g., migrant and nonmigrant) was sought to enable the potential sampling of participants with different backgrounds and lifestyles for the purpose of comparative analysis. Other factors considered in the selection of sites were accessibility to the area (in terms of distance, transport facilities, and safety and security) and the language spoken in the area (which was to be the same as the language of the researchers to avoid an additional level of translation). The selected geographical sites were West Shilmandi village in Bangladesh’s Norshingdi district, Bagga Sheikhan and Sultan Pura in Pakistan’s Rawalpindi district, and, in Nepal, Godawari and Badikhel Village Development Committees in Lalitpur district, as well as Lalitpur submetropolitan city.

### Interview, Recruitment, and Sampling

A core component of the approach to discovering grounded theory is theoretical sampling. With theoretical sampling, sampling decisions are driven by the emerging categories and developing theory and so the researcher jointly collects, codes, and analyses data, deciding what data to collect next and from where on the basis of its theoretical purpose (Glaser, 1978; Glaser & Strauss, 1967). To enable sampling to be theory driven in this multicountry study, we had a coordinating researcher (Bethan Hatherall) whose role it was to oversee the research process, to lead on the coding and theory development, and to communicate sampling decisions to the research teams in each of the three countries.

Prior to commencing formal data collection and as part of the local research teams’ training, the teams familiarized themselves with the study sites, employed rapid appraisal techniques, and facilitated informal group discussions. This process informed the decision to include the broad and flexible categories of people with TB, family members, and health workers in our sample; the development of the interview guides; and the sampling criteria for the first few interviews. Consistent with previous research on TB in South Asia (Atre et al., 2011; Baral et al., 2007; Ganapathy et al., 2008; Liefooghe et al., 1995; Nair et al., 1997; Sagili et al., 2016; Somma et al., 2008), the negative impact of TB on marriage and marriage prospects, particularly for women, was raised by participants of these informal group discussions, and so the first stage of sampling for the individual interviews involved recruiting both married and unmarried men and women with TB. To enable subsequent theoretical sampling, an interview or a small number of interviews were translated into English, analyzed, and discussed prior to further data collection. As well as informing decisions on who to interview next, the analysis also generated ideas to be explored in subsequent interviews, relating to, for example, spousal roles and desirable characteristics of a bride and groom. Initially, participants were recruited primarily with the assistance of community- or clinic-based health workers, but as the research progressed, research participants themselves assisted in identifying other potential participants with the attributes we sought.

The interview guides used for people with TB and health workers were based on a series of general questions that were similar across all three countries to ensure comparability. The interviews with people with TB primarily sought to encourage them to describe their personal experiences, from first symptoms or suspicions onwards, while the interviews with health workers sought to explore the nature of their role, the terms they use for TB, their thoughts on how patients experience TB and its impact, perceived reasons for delayed treatment seeking and nonadherence to treatment, their own fears of TB, and their ideas for improving the experiences of TB patients. For the interviews with family members of people with TB, vignettes, which are short stories depicting hypothetical characters and scenarios to which the research participants can relate, were developed. By inviting participants to respond to the vignettes, beliefs, perceptions, opinions, and attitudes are elicited (Barter & Renold, 1999). By beginning the interviews with vignettes, it was left to the person being interviewed to disclose, or otherwise, that someone in his or her family had TB. Once he or she did disclose, he or she was encouraged to continue talking about his or her personal experiences of having a family member with TB.

In total, we conducted 73 individual interviews between April 2008 and February 2009: 48 with people with TB (25 women, 23 men), 15 with family members of people with TB (11 women, four men), and 10 with health workers (six women, four men). Of the 48 people with TB interviewed, ages ranged from 16 to 66 years and included 10 women and eight men who were unmarried. Each interview took approximately 45 to 60 minutes, was conducted in Nepali, Newari, Bengali, Urdu, or Punjabi by members of the local research teams, and was audio recorded with informed consent.

### Analytical Strategy

All interviews were translated into English by the local research teams who were able to address issues of dialect and locally idiomatic phrases and consider cultural understanding (Chidarikire, Cross, Skinner, & Cleary, 2018). The translated transcripts were then read and coded by the coordinating researcher who had regular discussions via telephone and email with the research teams about the research process, including the analysis. In addition, for each interview, a document was produced summarizing the background of the participant (including, for example, age, personal circumstances, and TB history), the substantive findings with narrative descriptions, and relevant verbatim quotes and notes on theoretical ideas evoked by the interview data. To check consistency of understanding of the translated interviews, samples of these summary documents were sent to the research teams in the three countries for comments. Furthermore, in all three countries, a sample of the full interviews was analyzed separately in the original language by the research teams and in translation by the coordinating researcher, and the analyses compared. Both the sharing and comparing of analyses was especially useful as a way of identifying where translation may have affected meaning and interpretation.

Once all the interviews for a site had been completed, the summary documents were uploaded into NVivo 7 and recoded using both the codes previously used on their corresponding transcripts and any relevant additional codes and analytical categories developed subsequently. These documents were reread and notes were made of developing theoretical understanding within and across sites, with the full transcripts revisited as necessary. Based on these notes, vignettes were developed and used as a basis for discussion in focus groups to refine our developing theory.

### Supplementary Focus Groups

Eight audio recorded focus group discussions, each lasting approximately 1 hour, were conducted across two sites in Nepal and one site in Pakistan. Time and capacity constraints prevented us from conducting focus groups in the other two sites. Of the eight groups, five (three with women and two with men) comprised people living in the local communities and three (two with women and one with men) were comprised specifically of people with TB. Vignettes were developed, which incorporated the theoretical ideas developed from the analysis of the interview data. Such ideas related to, for example, spousal roles and expectations, age at marriage, and prior family approval of a marital union. The participants actively discussed the scenarios, drawing on their personal experiences to theorize as to why a vignette character might think, feel, or act in a certain way. Much like theoretical group interviews (Morse, 2007), the discussions enabled us to confirm, challenge, and enhance the analysis of the interview data and the theories generated in the process, and to evaluate their verisimilitude with the views and experiences of those with TB and other community members. The audio recordings were translated and coded and incorporated into the analytical notes from the interviews.

## Findings

Across all three countries, for parents especially, ensuring their children marry was found to be a matter of great concern regardless of whether they or their children had TB. While TB exacerbates this worry, the data suggest that worry is exacerbated not by TB per se, but by illness in general.

*People hide their diseases. [. . .] They hide disease to get a good proposal for their daughter. That is why they hide. [. . .] They hide all types of diseases*. (Female focus group participant, Pakistan)

However, not all diseases are likely to affect marriage prospects equally and indeed the marriage prospects of all those with TB are not affected equally either. Participants spoke of the detrimental impact of TB on marriage prospects being far greater for women than for men.

*If someone hears that an unmarried girl has TB, there would be a problem with her marriage. Who will marry a diseased girl?* (Man with TB, Bangladesh)

Through the interwoven processes of data collection and analysis to understand participants’ reasoning, we sought to build a theoretical understanding of the relationships between TB, marriage prospects, and gender in the contexts of the research sites. Contextualized gendered roles and responsibilities are at the center of this theoretical understanding.

### A Hardworking Wife

An unmarried man with TB in Nepal said that society is harder on women not only if they have any disease, but also if they do not or are not able to work hard. The impact of TB on a person’s ability to work, and in particular on a woman’s ability to work, may be as much of an issue as TB itself. This idea was supported across the interviews and is illustrated by the following quote.

*With TB people become weak. They have difficulties in doing their work. People say it is better to have no relations with them*. (Man with TB, Pakistan)

In the research sites, marriages are often arranged between families. Indeed, arranged marriages represent at least 95% of all marriages in Bangladesh and Pakistan, and are customary in Nepal also (Allendorf & Ghimire, 2013; Gabriela, 2014). While participants said that being healthy and hardworking are characteristics sought in both a bride and a groom, they emphasized the particular importance of a groom’s financial status.

*If a boy is not financially strong then no one will consider him [for marriage], but if a boy is financially strong then [they] will*. (“Lady Health Worker,” Pakistan)

The financial strain and consequences of TB emerged as a prominent theme throughout the interviews and focus groups. However, in all five study sites, it is customary after marriage for a woman to join her husband’s household, living with his parents and sometimes his siblings too, and so “financial strength” may be a family characteristic rather than an individual characteristic. Therefore, even if a prospective groom is unwell and unable to work, this may be compensated for by the status of his family and the ability of other family members to provide an income.

To understand why being hardworking is deemed particularly important in a prospective wife, it is necessary to consider the role and responsibilities of a wife in the contexts of the research sites. A married woman interviewed in Pakistan described her role as a wife as being to “*take care of my home, my husband, [. . .] give him breakfast, do the washing, take care of my kids, fulfill their needs.*” Another married woman in the same site in Pakistan described how this role had been jeopardized by her TB diagnosis and said that her husband had been advised to leave her as a result. When asked what she thought would have happened if their situations had been reversed, she said she thought she would have been advised to stay and take care of him.

*[A wife] has a lot to do at home by herself. People otherwise taunt her that she is sick. [A man is not taunted in this way] as the household belongs to him.* (Married woman with TB, Pakistan)

If a woman is unable to work because of illness, this not only makes her unable to fulfill her role as a wife, but, living in her husband’s family’s household, may also result in a greater workload for her mother-in-law and, until they get married, any unmarried sisters-in-law. It is therefore in the interest of not just the husband, but also his family, that he has a healthy, hardworking wife.

*Some [families] may need a daughter-in-law who can do household activities. An ill person cannot do such work.* (Female focus group participant, Nepal)

In Bangladesh, a perception that abstinence from food preparation is necessary when a person has TB may further compound the problem for women.

*[If a woman has TB] there will be more problems. They can’t cook, they are not allowed to cook. Even they are not allowed to go to the kitchen sometimes.* (Married man with TB, Bangladesh)

While in the interviews conducted in Nepal and Pakistan no reference was made specifically to the need to abstain from food preparation, emphasis was given to not sharing food and eating utensils for the prevention of TB transmission. As Try (2006) writes in her article on leprosy stigma within the Maithili ethnic group in Nepal, restrictions over the daily activity of food preparation affect women more than men, as the socially expected role of women is located within the domestic sphere.

### A Healthy Mother

Across all three countries, some of those interviewed expressed concerns over the potential risks of TB to a pregnancy or an infant. As a result a TB diagnosis can be seen as particularly problematic for a woman who is pregnant or who is wanting or expected to become pregnant, such as a soon-to-be or newly married woman.

*Born from that person with TB [a baby] can get TB [. . .] because the baby is in the womb of an infected mother who already has TB [. . .] If at that time that baby does not get [TB] then it may be affected in later life.* (Women with past TB, Nepal)

Indeed, untreated TB in pregnancy has been associated with poor obstetric and perinatal outcomes, including premature birth, low birth weight, and perinatal death (Getahun, Sculier, Sismanidis, Grzemska, & Raviglione, 2012; Marais, Gupta, Starke, & El Sony, 2010). Very rarely, the fetus can be infected in utero via the umbilical cord, but the greater risk of infection is after birth if the mother has untreated infectious pulmonary TB or if she has been taking treatment for less than 2 weeks (Ormerod, 2001). However, the data from this research suggest there are still concerns about the transmission of TB from mother to child, even if the mother’s TB is not thought to pose a risk to adults. This is because of the research participants’ perceptions of heightened vulnerability of infants and children to TB infection across all sites, which is confirmed by the WHO (WHO, n.d.). A research participant in Nepal said that her mother-in-law, who had been diagnosed with extrapulmonary TB, had been told by a health worker that she was not infectious, but that she should limit contact with her grandchildren anyway as a precaution. Although this was the experience of a grandmother, it seems plausible that concerns about the particular vulnerability of infants and children may contribute to concerns about marriage to (and having children with) a woman with current or indeed treated TB.

The developing overarching idea that the social consequences of TB relate to TB’s disruptiveness to gendered roles and responsibilities (such as those of wife and mother) informed the purposeful sampling decision to specifically include people with TB who have young children. One of these had been diagnosed with TB during pregnancy and her experiences highlight just how disruptive TB can be, not just to the role of wife, but also to becoming and being a mother. She said that she had been diagnosed just after giving birth and that her immediate admission to a sanatorium for treatment meant that she had not been able to see her baby for the first month of its life. Even once discharged, she was forbidden physical contact with her baby until completion of her 9-month course of treatment, during which time it was cared for by her mother-in-law.

### For Whom and in What Circumstances?

Having developed explanations as to why TB impacts on marriage prospects and why the impact is anticipated to be greater for women than for men, the explanatory theory will be developed further by considering variation in the anticipation of a negative impact of TB on the marriage prospects of women. For which women, and in which circumstances, does a TB diagnosis reduce marriage prospects? From the data, a number of factors that appear to safeguard marriage prospects were identified. These include having characteristics highly desired in a spouse (other than good health and the ability to work hard), which compensate for the TB, and, associated with this, family approval of a marriage, having multiple potential marriage partners, and young age.

A woman with TB living in an urban area in Pakistan and studying to become a nurse had received a proposal prior to her diagnosis and said the marriage was still likely to go ahead, reasoning that her education and future earning potential were highly valued, outweighing the negative implications of her ill-health.

*If a girl is doing a job then there is no problem. If that girl is just a housewife then there can be many problems. Nowadays everyone wants a daughter-in-law who earns money.* (Woman with TB, Pakistan)

In Nepal, one of the female focus group participants said that if a groom-to-be likes and especially loves the bride-to-be he will think the illness is curable and will marry her anyway. However, in contexts where marriages are often arranged between families, emotional ties between a couple do not invariably make marriage more likely in the face of a TB diagnosis. Indeed, some of those interviewed in Pakistan and Bangladesh anticipated that if the marriage had already taken place, there would be tension between the new wife and her in-laws, and the potential for divorce, regardless of whether the wife is loved by her husband. A volunteer health worker interviewed in Bangladesh told of a man who requested that she not disclose his wife’s TB to his family members, that she not come to their home, and that he collect his wife’s treatment weekly rather than his wife having to do so daily, so as not to arouse suspicion. He was particularly worried that his mother might ask his wife to leave if she found out about the TB.

The importance of family approval of a marriage is highlighted in a story told by a female community health worker, known as a Lady Health Worker, in Pakistan. She described a case she knew of where a man married a woman away from his village and returned with her to his family. After just 1 month of marriage, she was diagnosed with TB and he divorced her. On the face of it, it appears the divorce was due to her TB or at least her ill-health, but the man had married for love without his parents’ approval, and following her illness and confirmed diagnosis his parents insisted that they divorce. Her confirmed poor health provided them with a legitimate reason to insist on the dissolution of a marriage of which they already disapproved.

*His mother said “You have brought an ill girl from Lahore. Who told you to marry out of this family?” That is why she was divorced. [. . .] In spite of [knowing TB is curable] they forced their son to divorce his wife because it was a love marriage.* (“Lady Health Worker,” Pakistan)

The disapproval for marrying “out of this family,” expressed in the above quote, refers to the custom of consanguineous marriage. Consanguineous marriage is practiced in all three countries included in this study, although not among the ethnic groups, including Newar and Hindu castes to which the research participants in the sites in Nepal belonged (Subedi, 2001). While participants from the sites in Bangladesh and Pakistan emphasized good health and being hardworking as desirable characteristics in a bride or groom, being from within the extended family was seen as equally if not more important. A cousin with TB may be a more desirable prospective bride (or a niece with TB may be a more desirable daughter-in-law) than a woman without TB who is unrelated.

*My in-laws have this concept that first of all they want the girl to be from their own family. Obviously they also check that the girl is in good health.* (Female focus group participant, Pakistan)

Marriage prospects may also be greater, and the impact of TB therefore less, if a woman (or indeed man) has many potential eligible marriage partners. For example, if, due to TB and its lengthy treatment, a woman’s marriage to her cousin does not go ahead, then it is not her last opportunity to marry if she has many other as yet unmarried cousins to marry once she is in good health again.

*Here it’s a family system where one individual has at least three or four proposals. [. . .] in our family it doesn’t happen [that someone can’t get married] as we have four or five relations. For example, my wife is my cousin from my father’s side [ . . .].* (Married man with TB, Pakistan)

Finally, treatment of TB requires a 6-month or more course of drug therapy and participants generally considered it preferable to delay marriage until the course has been completed. In all five sites, it was evident that women are generally expected to get married at a younger age than men and that the optimum age range for marriage for women is lower and narrower than that for men, with the median ages at first marriage for women in Bangladesh, Nepal, and Pakistan being 19, 21, and 23 years, respectively, compared with 26, 23, and 26 years, respectively, for men (United Nations, 2017). A woman in her late teens or early 20s who delays marriage for 6 months or more because she is taking treatment for TB could find herself at an age considered too old for marriage. This idea, that the *length* of TB treatment could affect the marriage prospects of women, developed from an interview with a woman with TB in Nepal. She implied that she had married late at the age of 25 years because of an illness and the medical investigations and treatment required. Subsequent focus group discussions explored and confirmed the idea that for a woman in Nepal marrying at 25 years of age is considered late and would arouse suspicion, particularly if she was not engaged in education or employment.

*How I feel is that in the context of Nepal, women especially get married at a young age. [. . .] Now everyone speculates about the reason for not getting married until 25 years old, that there might be some problem.* (Male focus group participant, Nepal)

### Concealing and Disclosing TB

While the anticipated impact of TB on marriage prospects may be lessened if the woman has other desired characteristics (such as an education and earning potential, or, where consanguineous marriage is practiced, being from within the same extended family), in the absence of such characteristics, or of absolute confidence that these characteristics will compensate for TB disease, being able to keep a TB diagnosis confidential takes on particular importance. Unmarried women with TB in particular expressed concern or were reported to be anxious about the confidentiality of health services and their own ability to conceal their diagnosis from others. Difficulties in accessing treatment confidentially are thought to be particularly pronounced for women, as illustrated by the following quote from a woman with TB in Bangladesh:

*In the case of men, they can easily go for medicine to the health center. No one asks them. But when any girl goes to the health center, everyone asks why, why has she gone there, what is her problem? It is very embarrassing.* (Woman with TB, Bangladesh)

In Nepal, a woman with TB pointed out that, while there is variation from family to family, a woman is usually asked where she is going, with whom, when she will be back, and so on, while a man is freer to move around without being questioned. Indeed, in Pakistan, participants said that it is expected that a woman be escorted to a health facility by her husband or a male relative. However, even in the absence of such an expectation, discrepant literacy rates between men and women may make being escorted a necessity. According to a man interviewed who was living in a rural area in Nepal, women cannot go to the health facilities on their own as they are often illiterate.

*How can we send a female alone? She can’t read so wherever she goes she needs a male along with her.* (Man with past TB, Nepal)

TB can be particularly difficult to conceal because of the need to access health services frequently over the lengthy duration of treatment. At the time of data collection, Directly Observed Treatment (DOT), an approach in which all patients are required to attend the health clinic daily to take their drugs, was a core component of the global Stop TB Strategy (WHO, 2006), and hence of almost all national TB strategies. Furthermore, health workers themselves may divulge the diagnoses of their patients. This is particularly likely, according to a health worker living in a rural area in Pakistan, if the health worker lives in the same village as the patient.

*If the health worker knows then everybody will come to know about this.* (Male health worker, Pakistan)

While a man with TB spoke positively about how during holidays, when the clinic is shut, the health worker comes to his home to give him his treatment, a woman with TB living in a rural area of Nepal described how she was terrified when the health worker came to her home that he might disclose her TB to her husband and neighbors.

As the theme of disclosure emerged strongly from the analysis of the interviews with people with TB and their family members, we asked in the interviews with health workers and TB volunteers how the responsibility for tracing patients who have not attended clinic appointments (another component of the global Stop TB Strategy at the time) is managed alongside the patients’ desire for confidentiality. A volunteer health worker interviewed in Bangladesh was quite clear that the patient’s right to confidentiality is not as great a priority for her as protecting the public’s health by ensuring adherence to treatment.

*Actually patients should handle their family problems. I’m responsible for ensuring DOT is adhered to for the sake of other community members.* (Volunteer health worker, Bangladesh)

Likewise, in Nepal, a TB volunteer interviewed, whose role included tracing patients who had not come for treatment, did not deem confidentiality of the patient’s TB status to be important and strongly felt that people with TB should disclose, both for their sake and for the sake of others. While some were sensitive to the patients’ needs and desires for confidentiality, the need to ensure TB treatment adherence was clearly prioritized.

## Discussion

At the center of our explanatory theory is the valuation of a prospective wife or daughter-in-law on the basis of her health and her ability and willingness to work hard. More than 50 years ago, in his sociological book on stigma, Goffman (1963) argued that no attribute is inherently stigmatizing, but that instead a number of factors influence whether a person with a potentially stigmatized attribute experiences stigma. These factors were expanded upon by Jones et al. (1984) and referred to as dimensions of stigma. Of the dimensions, it is *disruptiveness* (essentially the same as Goffman’s *obtrusiveness*) that comes to the fore in the explanatory theory of TB’s impact on marriage prospects presented in this article. Disruptiveness refers to how much an attribute or condition, such as TB, interferes with “normal” life and social interactions, and the nature of the disruptiveness influences the extent to which others can overlook what Goffman (1963) refers to as the perceived “failing” (p. 66). In the contexts of the research sites where a woman moves into her husband’s household after marriage and is expected to take care of her husband, his family, and the home (relieving her mother-in-law of responsibilities), the ability to work hard (and to take care of others rather than to be taken care of by others) is central to fulfilling the socially expected role of wife. As Allendorf and Ghimire (2013) write of marriage in Nepal, a married woman is expected to devote herself to caring for her husband and the rest of the family. Physical illness and the additional practical difficulties for women of accessing treatment (relating to, for example, restrictions on movement and illiteracy) interfere with this generally, while (widely required but unnecessary) restrictions on food preparation and contact with shared eating utensils interfere with the ability to work hard in the domestic sphere specifically. Furthermore, concerns over the potential risks of TB and TB treatment during pregnancy, and the perceived heightened vulnerability of infants and children to TB infection, make TB particularly problematic for women wanting or expected to become pregnant, such as soon-to-be or newly married women.

Leary (2001) argues that stigma involves a person being devalued on the basis of his or her possession of attributes that are “consensually regarded as legitimate criteria for rejection” (p. 7). TB’s disruptiveness comes to the fore in our explanatory theory of TB’s impact on marriage prospects, but it is specifically TB’s disruptiveness to the socially expected gendered roles of wife and mother, which makes it a characteristic consensually regarded as legitimate grounds for rejection. Consider the story told by the Lady Health Worker in Pakistan of the man who had married a woman from outside his family and without his parents’ approval. The woman’s initial “failing” (to use Goffman’s term), that she was not a cousin, could be overlooked because it did not conflict with her ability to fulfill her role as a wife, and so did not in itself constitute a sufficiently legitimate reason for his parents to insist on the dissolution of the marriage. Her subsequent diagnosis with TB, however, did. It was a “failing” that could not be overlooked because it conflicted with her ability to fulfill her role as a wife. Goffman (1963) states that the attributes that are discreditable, in other words most likely to be associated with stigma, are those that are not congruous with society’s view of what a particular type of individual should be. In the contexts of the three South Asian countries, for some women in certain circumstances TB is incompatible with what is socially expected of them as wives (or daughters-in-law) and mothers. Lessening this incompatibility needs to be central to approaches to reducing the negative impact of TB on marriage prospects.

From a realist perspective of causality, this mechanism is only meaningful as a causal explanation if the context in which it becomes generative is understood. Providing some of the sociostructural context are factors, such as women joining their husband’s household after marriage and the role of wife being predominantly located in the domestic sphere. Such factors suggest opportunities for addressing the impact of TB on marriage prospects, such as promoting education and employment among women, to extend women’s roles beyond the domestic sphere. While these may seem beyond the usual remit of TB Programs, firmly within their remit are approaches that reduce the disruptiveness of TB to current gendered roles, thereby reducing the significance of TB to the valuation of a prospective spouse.

Health and the ability or willingness to work hard are valued characteristics of a prospective wife or daughter-in-law. TB jeopardizes these characteristics, but less so when it is detected early and then treated promptly and effectively. Improving case detection and treatment, particularly among unmarried and newly married women, is therefore expected to contribute toward reducing TB’s impact on marriage prospects. While National TB Programs are already working toward increasing early case detection and delivering prompt and effective treatment, it seems crucial that, given the strong gender dimension of this manifestation of TB stigma, the implications for women and men, both married and unmarried, are carefully and continually considered. This is known as mainstreaming gender, which the United Nations (1997) defines as “the process of assessing the implications for women and men of any planned action, including legislation, policies or programs, in all areas and at all levels”.

The approach at the time of data collection to TB treatment delivery known as DOT, which requires daily visits to the clinic for at least the first 2 months of treatment, poses difficulties for those with TB, but these difficulties vary in degree and form according to individual circumstances and gender. In this study, participants reported that women have less mobility, as their movements are questioned and arouse suspicion, and less autonomy, as they sometimes require or are required to have an escort to the clinic. Similar findings have come from research in neighboring India. Fochsen et al.’s study on the doctor–patient relationship in a rural district found that a woman with TB would normally be accompanied to the doctor by her husband, in-laws, or parents and is perceived as being dependent and passive (Fochsen, Deshpande, & Thorson, 2006). Ogden et al. (1999), writing about gender and TB in India, point out that “younger women have relatively junior status in their households and communities, less mobility, less autonomy and greater constraints in accessing resources for treatment” (p. 857). Even though the current global End TB Strategy (WHO, 2015) has a reduced focus on DOT, it persists in many countries.

Following a gradual shift in rhetoric, and even practice, away from service-centered care (Lienhardt & Ogden, 2004; Zachariah et al., 2012), the global End TB Strategy has as the first of its three core components patient-centered care (WHO, 2015). However, as this article shows, patient-centered care must not be centered around a single type of (male, married) patient. This requires both gender mainstreaming and meaningful involvement of service users in service design to ensure that “improved” models of treatment provision do not simply substitute difficulties of access relating to availability and affordability of care with difficulties of access relating to the acceptability of care for unmarried and newly married women. As presented earlier, while a home visit by a health worker was acceptable to and appreciated by a male participant with TB living in an urban area of Nepal, such a visit was not acceptable to a female participant with TB living in a rural area, as she was terrified that the health worker would disclose her TB to her husband and neighbors. Furthermore, (unnecessary) advice given by some health workers and TB volunteers to abstain from food preparation, to not share eating utensils, and to limit contact with children, as reported by research participants, has a disproportionate impact on women and contributes to creating a dissonance between TB and the gendered roles of wife and mother.

With few documented and evaluated TB stigma interventions, Courtright and Turner (2010) suggest that, as with HIV and mental illness, the most promising approach to reducing TB stigma may be to empower those with TB to resist “stigmatizing external judgments” (p. 38). However, another approach is to empower those with TB not just to resist but to avoid what Pescosolido (2015) refers to as “disclosure carryover,” meaning the risks associated with disclosure. Related to TB’s anticipated detrimental impact on the marriage prospects of women, participants reported a greater desire among women to conceal a TB diagnosis and greater anxiety over the confidentiality of their condition. An understanding of how current TB policies and services influence what Goffman (1963) terms the *evidentness* and Jones et al. (1984) term the *concealability* of a person’s TB status will highlight opportunities for improving the design and delivery of services in such a way that people with TB (both male and female, married and unmarried) are given greater control over managing knowledge of their condition and therefore of any manifestations of stigma associated with it. Because of the structure of health clinics in low-income countries and the need to access TB services frequently over a lengthy period, it can be difficult for both men and women to keep their TB confidential; and for young women, having less mobility and autonomy compounds the problem. As part of the shift from service-centered to patient-centered care, discussion and consensus are needed on the position of confidentiality in TB care, as currently it is a priority for those with TB but not always for those responsible for providing TB care.

Both a strength and challenge of our research was conducting comparative analysis and theoretical sampling across sites in multiple countries. This was made possible by having a coordinating researcher whose role it was to oversee the research process, manage translation, conduct coding, and communicate emergent theory and theory-driven sampling decisions. Collecting qualitative data from multiple geographically disparate sites certainly aided comparative analysis and this enabled the development of a causal theory that is grounded and local, but which also has scope beyond a single setting.

The disruptiveness of TB to the roles of wife (or daughter-in-law) and mother, can both explain why, if unmarried, a woman with TB may be considered less favorably as a prospective wife or daughter-in-law, and why, if married, a wife may anticipate being or be threatened with divorce, as has been reported in previous studies (Liefooghe et al., 1995; Long, Johansson, Diwan, & Winkvist, 2001). Similar disruptiveness to gendered roles can also arise with diseases other than TB, as highlighted both by the research participants and by previous studies that have associated leprosy and thalassemia with reduced marriage prospects, particularly for women, in Asia (Chattopadhyay, 2006; Sermrittirong, van Brakel, Kraipui, Traithip, & Bunders-Aelen, 2015; Try, 2006). There is likely to be a thread running through the relationships between disease and the (de)valuation of a prospective spouse, marital tensions, and divorce that cannot be fully appreciated when these relationships are explained in isolation of each other and in relation to just one disease category, suggesting opportunities for developing the theory further. Theory is after all, as Glaser and Strauss (1967) emphasize, not a perfected product, but “an ever-developing entity” (p. 32). Nevertheless, given the dearth of evidence on effective interventions to reduce TB stigma (Sommerland et al., 2017), the explanatory theory presented in this article provides a much needed theoretical basis upon which to consider policy and program responses to TB’s adverse impact on marriage prospects as a dimension of TB stigma.
